# Prenatal Cannabinoid Exposure: Emerging Evidence of Physiological and Neuropsychiatric Abnormalities

**DOI:** 10.3389/fpsyt.2020.624275

**Published:** 2021-01-14

**Authors:** Mina G. Nashed, Daniel B. Hardy, Steven R. Laviolette

**Affiliations:** ^1^Department of Anatomy and Cell Biology, University of Western Ontario, London, ON, Canada; ^2^Department of Physiology and Pharmacology, University of Western Ontario, London, ON, Canada; ^3^Department of Obstetrics & Gynecology, University of Western Ontario, London, ON, Canada; ^4^Department of Psychiatry, University of Western Ontario, London, ON, Canada

**Keywords:** cannabis, marijuana, THC, neurodevelopment, prenatal, pregnancy, placenta, cannabinoid

## Abstract

Clinical reports of cannabis use prevalence during pregnancy vary widely from 3% to upwards of 35% in North America; this disparity likely owing to underestimates from self-reporting in many cases. The rise in cannabis use is mirrored by increasing global legalization and the overall perceptions of safety, even during pregnancy. These trends are further compounded by a lack of evidence-based policy and guidelines for prenatal cannabis use, which has led to inconsistent messaging by healthcare providers and medically licensed cannabis dispensaries regarding prenatal cannabis use for treatment of symptoms, such as nausea. Additionally, the use of cannabis to self-medicate depression and anxiety during pregnancy is a growing medical concern. This review aims to summarize recent findings of clinical and preclinical data on neonatal outcomes, as well as long-term physiological and neurodevelopmental outcomes of prenatal cannabis exposure. Although many of the outcomes under investigation have produced mixed results, we consider these data in light of the unique challenges facing cannabis research. In particular, the limited longitudinal clinical studies available have not previously accounted for the exponential increase in (-)-Δ9– tetrahydrocannabinol (Δ9–THC; the psychoactive compound in cannabis) concentrations found in cannabis over the past two decades. Polydrug use and the long-term effects of individual cannabis constituents [Δ9–THC vs. cannabidiol (CBD)] are also understudied, along with sex-dependent outcomes. Despite these limitations, prenatal cannabis exposure has been linked to low birth weight, and emerging evidence suggests that prenatal exposure to Δ9–THC, which crosses the placenta and impacts placental development, may have wide-ranging physiological and neurodevelopmental consequences. The long-term effects of these changes require more rigorous investigation, though early reports suggest Δ9–THC increases the risk of cognitive impairment and neuropsychiatric disease, including psychosis, depression, anxiety, and sleep disorders. In light of the current trends in the perception and use of cannabis during pregnancy, we emphasize the social and medical imperative for more rigorous investigation of the long-term effects of prenatal cannabis exposure.

## Introduction

While global cannabis usage has been increasing for decades (1), more recent emphasis on the medicinal use of cannabis, and a liberalization of the political environment around cannabis, have contributed to shifts in regulatory policies. Following Uruguay, Canada became the second country to legalize the possession and sale of recreational cannabis at the federal level in October 2018 (2). Individual states in the US are also increasingly adopting more liberal recreational cannabis policies, despite illegal status at the federal level (3). It is, therefore, vital to emphasize the need for accelerated research in promoting an evidence-based approach to the rapidly changing policies and regulations regarding cannabis, particularly for sensitive subgroups, such as pregnant women.

Considerable evidence suggests that there is a fundamental lack of understanding among the general population regarding the potential risks of cannabis use during pregnancy. For example, in a recent anonymous survey from Hamilton, Ontario, an understanding that cannabis-derived phytochemicals, such as (-)-Δ9– tetrahydrocannabinol (Δ9–THC), can be transmitted to the fetus during pregnancy was insufficient in influencing the choice of whether to discontinue cannabis use while pregnant (4). These data are consistent with reports showing that, in the past two decades, the perception that cannabis use poses no risk during pregnancy has increased 3-fold among reproductive-aged women in both clinical settings and across large-scale nationally representative surveys in the US (5, 6). In particular, women who reside in areas where recreational cannabis is legalized and those who report regular cannabis use prior to pregnancy perceive far less risk of continued use during pregnancy, possibly owing to a positive perception of therapeutic effects and a lack of communication with health care providers regarding the risks (5, 7, 8). Indeed, in an online survey approximately half of the health care provider participants did not explicitly discourage prenatal cannabis use (9). This lack of perceived risk is reflected in the increasing rates of prenatal cannabis use. In North America, survey and toxicology data derived from large health care databases indicate that prenatal cannabis use increased by 62% from 2002 to 2014 (10), and by 170% from 2009 to 2016 (11). Prevalence of prenatal cannabis use also appears to be age-dependent: as low as 3% in women older than 34 years and as high as 22% in women aged 18–24 years (11), though self-reported prenatal cannabis use was as high as 35% in one relatively small sample (12). Importantly, data derived from self-reporting likely underestimates the prevalence of prenatal cannabis use due to social desirability bias, with at least one report illustrating a large disparity between self-reporting (2.6%) when compared to umbilical cord blood samples (22.4%) (13).

Several factors are related to the decision to consume cannabis during pregnancy. Self-reporting data often highlight the management of mood disorders, such as depression and anxiety, as primary reasons of prenatal cannabis use. This is consistent with data showing greater odds of cannabis use for pregnant women diagnosed with depressive and anxiety disorders (14), as well as those reporting stressful life events in the year prior to pregnancy (15). The management of nausea is another frequently reported reason for prenatal cannabis use (16). In one study, 83% of medically licensed cannabis dispensaries in Colorado recommended cannabis products to alleviate morning sickness, with the majority of recommendations based on personal opinion (17). Therefore, unlike the use of other illicit substances during pregnancy, there is a strong perceived medicinal incentive for the use of cannabis coupled with a lack of perceived risk, even among medically licensed dispensaries and health care providers. In the absence of rigorous scientific evidence and consensus on the effects of prenatal cannabis use, the aforementioned trends are, thus, likely to continue. In this review, we summarize recent clinical and preclinical data on the effect of prenatal cannabis use. In doing so, we consider neonatal outcomes, physiological effects, and neurodevelopmental outcomes. We also consider the strength of the available evidence and highlight areas of relative consensus and knowledge gaps. Summaries of the clinical and preclinical studies discussed in this review have been organized in Supplementary Table 1.

## Physiological Outcomes of Prenatal Cannabinoid Exposure

### Neonatal Outcomes

Meta-analyses and reviews of the literature have previously highlighted inconsistencies in the effects of prenatal cannabis exposure on neonatal outcomes including low birth weight (LBW), preterm delivery (PTD), and neonatal intensive care unit (NICU) admission (18–20). Notably, these analyses largely focused on studies dating from the late 1980's to the 2000's. Few of these studies provided information on gestational age of exposure and frequency of use, and none accounted for dose or concentration of Δ9–THC. This is particularly relevant given that the mean Δ9–THC concentration in cannabis has doubled over the past decade (21). Large cohort studies do suggest an association between *in utero* cannabis exposure and fetal growth restriction (FGR), including decreased head circumference (22). Additionally, early studies often did not delineate the effects of prenatal cannabis use from the impact of polydrug use. Several systematic reviews and meta-analyses have indicated that cannabis use leads to FGR and postnatal neurodevelopmental outcomes, however they are confounded by sociodemographic factors and the fact that users often used other drugs (e.g., tobacco) (18, 19, 23, 24). Indeed, more contemporary studies, some of which account for multiple factors, including *in utero* exposure to other drugs (tobacco, alcohol, benzodiazepine, and opioids), race, age, medical insurance, parity, and marital status, report that prenatal cannabis exposure alone is sufficiently predictive of LBW, PTD, and NICU admission (25–29).

In animal studies, Δ9–THC doses of approximately 3 mg/kg intraperitoneal (i.p.) (both acutely and chronically administered for 21 days) result in circulating concentrations of 8.6–12.4 ng/ml Δ9–THC after a 24-h washout period, which is consistent with that reported in cannabis smokers (13–63 ng/ml from a 7% Δ9–THC content cigarette) 0–22 h post inhalation, as well as in aborted fetal tissues (4–287 ng/ml) of pregnant cannabis smokers (30–32). In preclinical studies, which are better suited to control for environmental factors such as dosing and polydrug use, prenatal exposure to similar clinically relevant doses of Δ9–THC often recapitulate the LBW effect often reported in clinical studies (33–36). However, this effect is not always observed, with some studies reporting no effect on birth weight (37–42). This discrepancy may be related to route of administration, with LBW more often reported in studies that use i.p. injections, a lack of effect on birth weight more often reported in studies that use oral administration, and mixed results in studies that use vapor inhalation. In addition to the effects of prenatal Δ9–THC, studies are warranted to examine the safety of gestational cannabidiol (CBD; the major non-psychoactive constituent of cannabis) short- and long-term. Recent reports suggest that 62% of CBD users report pain, anxiety, and depression, all common ailments in pregnancy as reasons for use (43). In meconium and umbilical cord samples, both established markers of *in utero* cannabinoid exposure, the range of CBD is reported to vary from 10 to 335 ng/ml (44). Although there is a widespread perception that CBD is a “cure-all” to reduce these symptoms, its safety in pregnancy is unknown. Preclinical rodent studies are necessary to address the long-term effects of CBD on pregnancy and postnatal health.

Despite the higher quality of data in contemporary clinical studies, the independent and combined effects of Δ9–THC and CBD have not been delineated. This is a critical consideration given that the Δ9–THC:CBD ratios and concentrations can vary dramatically in available recreational cannabis products. In addition, CBD has been shown in clinical and preclinical studies to block or strongly mitigate the neuropsychiatric side-effects of Δ9–THC (45–47), meaning that high Δ9–THC/low CBD cannabis products may pose additional risks during prenatal development. Thus, while the preponderance of recent evidence suggests that prenatal cannabis use adversely impacts neonatal outcomes, a scientific consensus requires careful consideration of relevant variables such as polydrug use, the frequency and timing of prenatal cannabis use, and the relative chemical composition of the cannabis being consumed. Furthermore, preliminary correlational analyses highlight congenital outcomes, including cardiovascular defects, Down syndrome, and gastroschisis, which may be of importance for future investigation (48).

### Placental Abnormalities

CB_1_R and fatty acid amide hydrolase (FAAH), which hydrolyzes the endocannabinoid anandamide, are present in all layers of the human placenta (49). In rodent models, the ECS is present in midgestational placentas, where is has been demonstrated to play a critical role in placentation, trophoblast differentiation, as well as fetal outcomes, such as resorption rates (50). These findings highlight the importance of investigating the impact of exogenous cannabinoid exposure on placental development. The limited clinical data available demonstrate associations between prenatal cannabis exposure and increased placental weight (51), as well as enlarged umbilical vessel diameter (52). Closer examination in cultured human cells reveals that Δ9–THC hampers trophoblast remodeling through an antioxidant effect that prevents cell death of syncytiotrophoblasts (53). This is consistent with histological results from human placentas showing increased syncytiotrophoblastic knots and fibrin exudation in the villous stroma of cannabis users (34).

In rodents, prenatal Δ9–THC induces FGR with concurrent increases in placental weight and fetal to placental weight ratio (33, 36). Additionally, clinically relevant doses of Δ9–THC (3–5 mg/kg/day) lead to adverse morphological changes in placentas (34, 36). Specifically, Δ9–THC exposed animals exhibit an increase in labyrinth area (36), with increased diameters of trophoblastic septa (34). In pregnant mice given 5 mg/kg daily Δ9–THC, disordered structure of spongiotrophoblasts and decreased number of glycogen cells in junctional zone was also observed (34), although this effect was not recapitulated in rats exposed to 3 mg/kg daily Δ9–THC (36). Consistent with clinical findings of enlarged umbilical vessel diameter, maternal blood sinusoids within the labyrinth layer of exposed rats was found to be enlarged, while fetal blood space was reduced (36). Furthermore, labyrinth trophoblasts of exposed rats exhibited reduced glucose transporter 1 (GLUT1) and glucocorticoid receptor (GR) expression (36). Along with the abovementioned placental alterations, these findings implicate impaired maternal-to-fetal glucose transport as a possible mechanism of Δ9–THC induced nutrient insufficiency and FGR.

### Metabolic Outcomes

The mammalian endocannabinoid system (ECS) plays crucial regulatory roles in fetal peripheral organ development (54, 55). While the exchange of endogenous endocannabinoids between the mother and fetus is tightly regulated, approximately one-third of exogenous plasma Δ9–THC from the mother crosses the placental barrier to the fetus (56). The dysregulatory impact of sustained maternal administration of Δ9–THC on fetal metabolic processes is in the early stages of investigation. In rats, prenatal exposure to Δ9–THC [partial agonist of cannabinoid type 1 receptor (CB_1_R)] leads to decreased BW, brain to BW ratio, liver to BW ratio, and pancreatic weight at birth (35, 36). By 3 weeks of age, these offspring undergo postnatal catch-up growth resulting in glucose intolerance, paralleled by decreased pancreatic total and small islet density at postnatal day (PND) 21 and 5 months, specifically in female offspring (35). This is consistent with data demonstrating that endogenous regulation of CB_1_R is critically involved in fetal pancreatic islet organization (55). Moreover, activation of CB_1_R reduces pancreatic β-cell proliferation and impedes insulin receptor activity, while CB_1_R antagonism can improve insulin resistance (57, 58). Importantly, Δ9–THC exposed rats also exhibited reduced body weight and pancreatic weight at birth, suggesting that the commonly observed clinical outcome of LBW may be associated with fetal glucometabolic dysregulation, an outcome that may disproportionately impact the long-term metabolic health of female offspring (35). While the sexual dimorphism could be attributed to differences in circulating sex hormones, the concentrations of estrogen and testosterone were not different in Δ9-THC offspring, suggesting a potential epigenetic mechanism (35). Given the links between FGR and long-term metabolic disease (59), further studies are warranted to assess if any cardiometabolic defects manifest long-term.

At the cellular level, involvement of the ECS has been demonstrated in metabolic processes relevant to fetal development. Indeed, mitochondrial and endoplasmic reticulum (ER) stress contribute to gestational complications, such as FGR (60), and Δ9–THC has been shown to decreases oxygen consumption and membrane potential of rat heart mitochondria, an effect that appears to be independent of cannabinoid receptor activation (61). Similarly, in the brain, Δ9–THC impedes mitochondrial respiratory rate, both through CB_1_R and non-receptor-mediated mechanisms (62). In astroglial mitochondria, activation of CB1R hampers glucose metabolism and brain lactate production, leading to altered neuronal function and behavioral deficits (63). Recently, these effects were recapitulated in human placental BeWO trophoblast cells, where it was demonstrated that Δ9–THC treatment decreases mitochondrial respiration, as well as dose-dependently increases ER stress (64). These effects were blocked by CB_1_R/CB_2_R antagonism and underscore the importance of ECS homeostasis in the development of fetal energy homeostasis. Given that LBW Δ9-THC-exposed offspring exhibit postnatal catch-up growth, a driver of ER stress and mitochondrial dysfunction (65, 66), it remains possible that cannabinoids *in utero* could also *indirectly* influence the development and function of metabolic organs in postnatal life.

## Neurodevelopmental Outcomes of Prenatal Cannabinoid Exposure

### Cognitive Outcomes

Growing epidemiological and experimental evidence over the past two decades has demonstrated an association between cannabis use during adolescence (a critical period of neurodevelopment) and increased risk of cognitive deficits and neuropsychiatric disease (67–69). The ECS is also critically involved in fetal neurodevelopmental processes, including synaptic plasticity, as well as neuronal cell proliferation and differentiation (70, 71). Considering that Δ9–THC readily crosses the placental barrier from the mother to the fetus, these processes and their long-term cognitive outcomes are potentially vulnerable to disruption by *in utero* cannabis exposure.

To date, three large prospective longitudinal cohorts have been used to investigate the consequences of prenatal cannabis exposure on neurodevelopment: The Ottawa Prenatal Prospective Study (OPPS) (72–76), The Maternal Health Practices and Child Development Study (MHPCD) (77, 78), and The Generation R Study (GenR) (79, 80). These data highlight several cognitive and behavioral domains affected by *in utero* exposure to cannabis. Across childhood and adolescence, cannabis exposure was associated with deficits in memory, verbal reasoning, concentration, attention, and Bayley Scale of Infant Development (BSID) scores, as well as increases in hyperactivity, impulsivity, and aggression (81–83). At 10 years of age, exposure in the MHPCD cohort was also predictive of poorer academic achievement as measured by Wide Range Achievement Test—Revised (WRAT—R) reading and spelling scores (78). Additionally, functional magnetic resonance imaging (fMRI) on exposed subjects from the OPPS cohort showed altered executive function and visuospatial working memory processing into young adulthood (75, 76). However, most performance effects from these cohorts were relatively subtle, and inconsistencies were present. Indeed, a recent systematic review of these data and other smaller cohorts determined that outcomes differed on only 4.3% of cognitive measures (with cannabis exposure being associated with worse outcomes in 3.4% of cognitive measures) (84). This review also concluded that the statistical differences were not clinically significant. Importantly, however, these data are largely derived from the 3 large prospective studies, which were initiated between 1978 and 2001. Therefore, recent trends toward cannabis legalization, accompanied by increased frequency of use, and the sharp spike in Δ9–THC concentrations observed over the past two decades are largely unaccounted for in these analyses. Indeed, in a more recent retrospective observational cohort, a positive maternal Δ9–THC urine test at the first prenatal visit was associated with abnormal 12-month developmental scores in infants, as measured by the Ages and Stages: Social–Emotional Questionnaire (ASQ-SE) (27). Moreover, recent cross-sectional results from the ongoing Adolescent Brain Cognitive Development (ABCD) study, which recruited 11,875 children aged 9–11 years, found that prenatal exposure to cannabis was associated with deficits in attention, thought, and social problems after accounting for potentially confounding covariates (29). A moderate increase in the incidence of intellectual disability and learning disorders was also observed in a large retrospective analysis of children born between 2007 and 2012 in Ontario, Canada, though these results were not statistically robust (85). The cognitive impairments observed in longitudinal cohorts, though often subtle, are also corroborated by mechanistic plausibility.

For example, in a recent study, human induced pluripotent stem cells (hiPSC) induced toward neuronal commitment, thus mimicking developing fetal neurons, were exposed to Δ9–THC and CBD for 37 days (86). At the clinically-relevant doses studied, CBD produced neurotoxic effects, while Δ9–THC promoted precocious neuronal and glial differentiation, and induced abnormal functioning of voltage-gated calcium channels. Furthermore, *in utero* exposure to cannabis has been demonstrated to disrupt fetal cortical and hippocampal connectivity by activating CB_1_R-mediated degradation of proteins that stabilize microtubules, effectively limiting the computational power of circuits relevant to cognitive function (87). A specific loss of cholecystokinin (CCK) interneurons in the hippocampus has also been observed in mice prenatally exposed to Δ9–THC (88). Interestingly, when systematically compared, these effects were similar to those observed in animals perinatally exposed to alcohol (89). Changes in cognitive performance have also been observed in animal models of prenatal cannabis exposure. Adolescent and adult rodents prenatally exposed to either Δ9–THC or a synthetic CB_1_R agonist have been shown to exhibit impairments in learning, long-term memory, short-term olfactory memory, spatial working memory, and attention when compared to non-exposed rodents (37, 40, 90–92). Although most of this data was derived exclusively from male rats, one study that assessed both male and female offspring found a more pronounced cognitive deficit in males (40). Importantly, these cognitive deficits were associated with cortical changes including decreased glutamate and norepinephrine, increased kynurenine, and altered neuron morphology (37, 91–94). Cognitive deficits were also associated with decreased hippocampal glutamate and γ-aminobutyric acid (GABA) outflow and uptake, decreased CB_1_R expression, and impaired hippocampal long-term potentiation (LTP), a neurophysiological model for learning and memory (41, 90, 95).

### Neuropsychiatric Morbidity

Cognitive deficits are often symptomatic of neuropsychiatric morbidity, and the associated brain regions and neurophysiological pathways are often implicated in disease states including schizophrenia, depression, and anxiety (96). However, to date, there has been a paucity of longitudinal data specifically assessing the effect of *in utero* exposure to cannabis on the risk of developing neuropsychiatric disease. Of the large longitudinal studies, depression was only assessed in the MHPCD cohort, where it was found that exposure was associated with a higher rate of depression in adolescence (83). In the OPPS cohort, fMRI showed a correlation between *in utero* cannabis exposure and increased neuronal activity in bilateral prefrontal cortex (PFC) during response inhibition (97). This is of note since it has been demonstrated that neural processes involved in response inhibition are abnormal in schizophrenia (98). Indeed, recent data from the ABCD study was used to determine whether prenatal cannabis exposure was associated with psychosis proneness, as assessed by the Prodromal Questionnaire–Brief Child Version total score and psychotic-like experiences (PLEs) (29, 99). These analyses found that exposed children, ages 9–11, exhibited increased vulnerability to psychosis symptoms. Consistent with these findings, an analysis of children from the GenR cohort found that *in utero* cannabis exposure was associated with child psychotic-like experiences, assessed through the Youth Self Report questionnaire (100). In adolescence, *in utero* cannabis exposure was also linked to externalizing problems (aggressive/rule-breaking behavior) in three of the four large cohorts discussed: the MHPCD, the GenR, and the ABCD cohorts (29, 77, 101). Internalization problems (anxiety/depression features, such as withdrawal) were also observed in the MHPCD and ABCD cohorts (29, 77), whereas in the GenR cohort, internalization problems were associated with smoking cannabis prior to, but not during, pregnancy (101). Interestingly, these study found a similar associations with reports of paternal cannabis use during the pregnancy, which was interpreted by the authors as suggestive of a common etiology underlying both parental cannabis use and offspring psychotic-like experiences, in contrast with a direct *in utero* causal link between exposure and offspring phenotype (100, 101). Moreover, a recent study of live births in Ontario, Canada between 2007 and 2012, reported that prenatal cannabis use was associated with increased incidence of autism spectrum disorder in the offspring, though this analysis relied on self-reported retrospective data that may have suffered from underreporting of cannabis use and other residual confounding bias (85).

Molecular data from cannabis-exposed human fetal specimens have demonstrated a dose-dependent reduction of dopamine (DA) receptor subtype D_2_ in the amygdala basal nucleus, particularly in males, suggesting impairment in the mesocorticolimbic neural systems that regulate emotional behavior (102). Since tobacco co-use often occurs with cannabis use during pregnancy, it is also relevant to consider the combined effects of prenatal exposure to both of these substances. To this end, two studies have demonstrated that co-exposed infants and kindergarten aged children exhibit an attenuated cortisol response to stressors, with a greater effect observed in males (103, 104).

In support of the available clinical data, animal studies have further corroborated the association between *in utero* cannabis exposure and neuropsychiatric deficits. Early studies showed that prenatal exposure to Δ9–THC was associated with sex-specific alterations in the hypothalamus-pituitary-adrenal (HPA) axis (105). More recent data showed that PND12 rat pups prenatally exposed to Δ9–THC exhibited an increase in the frequency of ultrasonic vocalizations (USV) when removed from the nest, a behavior that is possibly analogous to human infant crying and that may indicate long-term neuro-behavioral alterations (39). When tested during adolescence and adulthood, these rats exhibited a decrease in play behavior and social interaction and an increase in anxiety-like behavior on the elevate plus-maze test (EPM), respectively. Consistent with these results, exposed adult males exhibited anxiety-like behaviors in another paradigm, the open field test (OFT), suggesting long-lasting behavioral effects of *in utero* exposure (38). The mesolimbic reward/motivation pathway may also be impacted by *in utero* exposure to Δ9–THC. Adult male and female rats prenatally exposed to Δ9–THC exhibited a dampened locomotor response to a challenge of amphetamine (40). DAergic neurons in mesolimbic pathway are involved in locomotor response to psychostimulants, such as amphetamines, suggesting a strong relevance of these behavioral outcomes to the risk of developing substance use disorders, which warrants further investigation. Indeed, early data demonstrated that exposed rats show increased self-administration of morphine, paralleled by an increase of μ opioid receptor density in the PFC, the hippocampus CA3 area, the amygdala posteromedial cortical nucleus, the ventral tegmental area (VTA), and the periaqueductal gray matter (106). Although in this study alterations were observed in exposed female, but not male, offspring, others have demonstrated an increase in the rewarding effect of morphine in exposed offspring of both sexes, with a stronger effect in males (105, 107). Many of these observed neuropsychiatric deficits may also be linked to the neurophysiological alterations discussed earlier in the context of cognitive deficits, as the brain regions (PFC and hippocampus) and neurotransmitter systems involved are commonly implicated in neuropsychiatric illness as well.

### Sleep Disturbances

Studies into the role of the ECS in sleep, as well as the potential for cannabis to alleviate symptoms of sleep disorders have suggested a role for the ECS in circadian regulation (108, 109). In the MHPCD cohort, neonatal electroencephalogram (EEG) analysis found that prenatal exposure to cannabis was associated with increased motility and disruptions in sleep and arousal (110). These disturbances persisted at 3 years of age, with exposed children exhibiting increased nocturnal arousal, more awake time after sleep onset, and lower sleep efficiency (111). However, these studies included a relatively small sample size, with 55 newborns initially assessed and 48 children assessed at 3 years of age, including non-exposed controls. More recently, child sleep outcomes were assessed in the ABCD cohort using 11,875 exposed children ages 9–10 (112). This study also controlled for multiple covariates, including mother's education, household income, parental marital status, race, and child sex. The investigators found that maternal report of cannabis use was significantly associated with symptoms of disorders of initiating and maintaining sleep, disorders of arousal, sleep wake disorders, disorders of excessive somnolence, and a summed sleep disorder score as measured by the Sleep Disturbance Scale for Children (SDSC) (112). Furthermore, children of mothers who reported daily use of cannabis during pregnancy were at increased risk of symptoms of disorders of excessive somnolence. These findings are highly suggestive of a long-lasting impact of *in utero* exposure to cannabis on circadian regulation, though further cross-study replication of these findings is needed. Additionally, there is a paucity of animal studies to address these effects and their possible neurophysiological mechanisms. Therefore, a causal link remains elusive and requires more controlled investigation.

## Paternal Cannabinoid Use and Epigenetic Considerations

To date, the large majority of studies examining long-term effects of exposomes in pregnancy have focused mainly on the maternal environment. As previously discussed, analysis from the GenR cohort revealed that paternal cannabis use was predictive of psychotic-like experiences and behavioral deficits in offspring at ages 7–10, independent of maternal cannabis use (100). Notably for this cohort, paternal cannabis use was derived from maternal reports, and was only determined for the pregnancy period, not prior to pregnancy. While the authors of this study hypothesized a potential common etiology underlying both parental cannabis use and offspring behavioral outcomes, it is also possible that paternal preconception use is causally associated with the observed offspring phenotypes. Recently, it was demonstrated in rats that Δ9–THC exposure during adolescence, prior to mating, may influence neurodevelopmental and behavioral outcomes in subsequent generations (113, 114). Offspring of Δ9–THC exposed parents, who were themselves unexposed, exhibited enhanced heroin self-administration paralleled by molecular and electrophysiological alterations in the striatum, a key component of the reward circuitry (113). Moreover, sex-specific effects were observed at the levels of gene expression and behavior (114). In terms of paternal cannabis use specifically, direct evidence now exists, in both humans and rats, demonstrating that Δ9–THC exposure alters DNA methylation in sperm cells (115). These alterations may represent a vector by which paternal toxicant exposure is able to influence genetic expression, and therefore development, in the offspring. Indeed, adult male offspring of premating Δ9–THC exposed fathers were shown to exhibit deficits in attentional performance and memory tasks, paralleled by alterations in acetylcholine signaling (116–118). Interestingly, both prenatal and adolescent exposure to THC has been shown to potently sensitize the brain's DA pathways, effects which persist into later life (119–121). Such Δ9–THC-induced dysregulation of mesocortical and mesolimbic DAergic transmission patterns may be critical biomarkers for not only increased addiction risks, but also an underlying mechanism linked to increased vulnerability to schizophrenia, mood and anxiety disorders. Notably, the evidence for the influence of paternal cannabis use on offspring outcomes is in the early stages and predominantly preclinical. While these studies provide an important case for biological plausibility and warrant further mechanistic exploration, clinical validation is vitally needed to parse the contributions of paternal and maternal cannabis use on offspring outcomes. Ideally, prospective investigations should, therefore, delineate offspring outcomes for paternal-only exposure, maternal-only exposure, and combined exposure from both parents.

## Conclusion

In this review, we presented a summary of the available data on the effects of prenatal cannabinoid exposure. With an emphasis on contemporary and emerging data, we considered the impact of prenatal cannabinoid exposure on neonatal outcomes, persistent metabolic and physiological disturbances, as well as neurodevelopmental and neuropsychiatric liability. We have also considered the emerging role of paternal and cross-generational effects of cannabinoid exposure. In human studies, the preponderance of evidence suggests that prenatal cannabinoid exposure is predictive of several adverse neonatal outcomes, most notably FGR and LBW. Physiological mechanisms that underly these abnormalities may also be associated with negative and persistent metabolic outcomes. The most recent data also suggests an association between *in utero* exposure to cannabinoids and cognitive, behavioral, and neuropsychiatric aberrations. Most notably, emerging evidence suggests an association between prenatal cannabinoid exposure and deficits in memory, attention, and learning. In addition, prenatal exposure is predictive of increased risk of depressive symptoms, prodromal symptoms of psychosis, and sleep disturbances. Importantly, these cognitive and neuropsychiatric aberrations appear early in development and are persistent into adolescence and early adulthood. Animal studies using cannabis constituents (Δ9–THC and CBD) have largely been consistent with the clinical observations, further providing possible mechanistic explanations. However, a consensus does not exist on many of these outcomes, largely owing to methodological limitations, some of which may be overcome. Notably, a shift from self-reporting to biological sampling would improve the quality of data collected in clinical settings, as would detailed analyses of the frequency of use and relative Δ9–THC dosing. Furthermore, considering some of the emergent sex-dependent effects discussed in this review, it is possible that early inconsistencies were confounded by a lack of sex-specific analyses, which should be a major consideration for future investigations. For example, among animal studies covered in this review, 62% only considered male offspring (see Supplementary Table 1). With many cannabis-based products now on the market, it is also important to delineate the effect of chemical constituents in cannabis, such as the effects of Δ9–THC vs. CBD, as well as the method of consumption (e.g., inhalation vs. ingestion). Concurrent with this, more animal studies are needed to better establish causal links and plausible biological mechanisms. Importantly, growing evidence points to the critical role of prenatal factors such as the health of the placenta, the effects of intra-uterine growth restriction, and other pre-natal complications impacting the downstream risk of developing various neuropsychiatric disorders. There is thus an urgent need to better understand the mechanistic links between these prenatal developmental events, their impact upon neurodevelopmental pathology and risk factors and how exposure to cannabinoids might synergistically modulate these complex interrelationships.

## Author Contributions

MN researched and wrote the manuscript and created the tables. SL and DH provided intellectual input and edited and wrote the manuscript. All authors contributed to the article and approved the submitted version.

## Conflict of Interest

The authors declare that the research was conducted in the absence of any commercial or financial relationships that could be construed as a potential conflict of interest.
