# Supplementary material for: Prenatal Cannabinoid Exposure: Emerging Evidence of Physiological and Neuropsychiatric Abnormalities
Source: Front Psychiatry. 2021 Jan 14;11:624275. doi: 10.3389/fpsyt.2020.624275 (PMC7841012; doi:10.3389/fpsyt.2020.624275)
Supplement: Supplementary file 1 [file Table_1.pdf]

Supplementary Material

Table S1- Descriptive Summary of Clinical and Pre-clinical Studies on Outcomes of Prenatal Cannabis Exposure Discussed in Review

**Clinical Studies**					
**Study**	**Cohort**	**Fetal & Neonatal Outcomes**	**Cognitive Outcomes**	**Neuropsychiatric Outcomes**	**Sleep Outcomes**
(Conner et al., 2016)	Meta-Analysis (31 studies)	No effects when controlling for tobacco use	N/A	N/A	N/A
(Gunn et al., 2016)	Meta-Analysis (24 studies)	↓ BW<br>↑ NICU Admission	N/A	N/A	N/A
(Singh et al., 2020)	Scoping Review (41 studies)	↓ BW in 3/7 studies<br>↑ NICU Admission in 4/6 studies<br>↑ PTD in 3/9 studies<br>↑ SGA in 5/10 studies	N/A	N/A	N/A
(English et al., 1997)	Meta-Analysis (5 studies)	↓ BW	N/A	N/A	N/A
(Bailey et al., 2020)	2 Cohorts (Appalachian, Rocky Mountain) (N=1,062)	↓ BW<br>↑ PTD<br>↑ NICU Admission<br>↓ Apgar score	N/A	N/A	N/A
(Kharbanda et al., 2020)	Large health system database (N=3,435)	↑ SGA<br>No effect on PTD or BW	↑ Abnormal 12-month ASQ-SE	N/A	N/A
(Nawa et al., 2020)	Boston Birth Cohort (N= 8,261)	↓ GA	N/A	N/A	N/A
(Carter et al., 2016)	Cape Coloured (mixed ancestry) pregnant women (N=103)	↑ Placental Weight	N/A	N/A	N/A

(Ortigosa et al., 2012)	Satellite of Meconium Project (N=225)	↑ Diameter of umbilical vessels	N/A	N/A	N/A
(Fried, 1980;Fried and Watkinson, 1988;Fried et al., 1998;Smith et al., 2006;Smith et al., 2016)	The Ottawa Prenatal Prospective Study (OPPS) (N=698)	↓ GA	↓ Memory (4 yo)<br>↓ Verbal scores (4 yo)<br>↓ Attention (6 yo)<br>↑ Hyperactivity (6 yo)<br>↑ Impulsivity (6, 9-12 yo)<br>↓ Visual perception (9-12 yo)<br>↓ Concentration (13-16 yo)<br>↓ Visual memory (13-16 yo)<br>↓ Verbal reasoning (13-16 yo)<br>↓ Medial, dorsolateral, ventrolateral PFC activity during working memory (fMRI; young adult)<br>↑ Left medial PFC activity during working memory (fMRI; young adult)	↓ Response inhibition (18-22 yo)<br>↑ Neuronal activity in bilateral PFC during response inhibition (fMRI; young adult)	N/A
(Scher et al., 1988;Day et al., 1991;Dahl et al., 1995;Goldschmidt et al., 2004)	The Maternal Health Practices and Child Development Study (MHPCD) (N=1,360)	↓ Birth length<br>↑ BW	↓ Mental (9 mos) Development<br>↓ BSID scores (9 mos)<br>↓ Verbal reasoning (3 yo)<br>↓ Short-term memory (3, 6 yo)<br>↓ Verbal reasoning (6 yo)<br>↓ Quantitative reasoning (6 yo)<br>↑ Impulsivity (6, 10 yo)<br>↑ Hyperactivity (6, 10 yo)<br>↓ IQ score (6, 10 yo)<br>↓ Abstract reasoning (10 yo)<br>↓ Concentration (6, 10 yo)<br>↓ Visual reasoning (10 yo)<br>↓ Learning and memory (10 yo)	↑ Depression (10 yo)<br>↓ Internalization (10 yo)<br>↑ Externalization (10 yo)	↑ Motility & disruptions in sleep and arousal (EEG; neonatal)<br>↓ Sleep efficiency (EEG; 3 yo)<br>↑ Nocturnal arousals (EEG; 3 yo)<br>↑ Wake-time after sleep onset (EEG; 3 yo)

(El Marroun et al., 2009;Jaddoe et al., 2012;Bolhuis et al., 2018;El Marroun et al., 2019)	Subset of The Generation R Study (GenR) (N= 9,778)	↓BW	↓ Attention (18 mos)<br>↑ Aggression (F only) (18 mos)	↑ PLEs (10 yo)<br>↑ Externalizing Problems (7-10 yo)<br>No effect on internalizing problems	N/A
(Fine et al., 2019;Paul et al., 2020;Winiger and Hewitt, 2020)	Adolescent Brain Cognitive Development (ABCD) Study (N=11,875)	↓BW	↓ Attention score (CBCL) (9-11 yo)<br>↓Thought score (CBCL) (9-11 yo)<br>↓Social score (CBCL) (9-11 yo)	↑ PLEs (9-11 yo)<br>↑Psychosis proneness (Prodromal Questionnaire–Brief Child Version total score) (9-11 yo)<br>↑ Externalizing Problems (9-11 yo)<br>↑ Internalizing Problems (9-11 yo)	↑ Disorders of initiating and maintaining sleep<br>↑ Disorders of arousal<br>↑ Sleep wake disorders<br>↑ Disorders of excessive somnolence<br>↑ Summed sleep disorder score (SDSC)
(Corsi et al., 2020)	Provincial birth registry (Ontario Canada, 2007-2012) (N=503,065)	N/A	↑ Intellectual disability & learning disorders (diagnosed after 4 yo)	↑ Incidence of ASD (diagnosed after 18 mos)	N/A
(Wang et al., 2004)	Human fetal specimens	N/A	N/A	↓ D_2 expression in the amygdala basal nucleus	N/A
(Eiden et al., 2020)	Prenatal clinics (N=238)	↓BW<br>No effect on PTD	N/A	↓ Cortisol response to stressors, esp. in M (cannabis + tobacco co-exposed) (2-36 mos)	N/A
(Stroud et al., 2020)	The Behavior and Mood in Babies and Mothers (BAM BAM) study (N=111)	No effect on GA, SGA, or Apgar score	N/A	↓ Cortisol response to stressors, esp. in M (cannabis + tobacco co-exposed) (1 mos)	N/A

Preclinical Studies							
**Study**	**Organism & Sex Studied**	**Drug Dose & Duration**	**Fetal & Neonatal Outcomes**	**Placental Outcomes**	**Metabolic Outcomes**	**Neuronal & Cognitive Outcomes**	**Neuro-psychiatric Outcomes**
(Benevenuto et al., 2017)	Mice<br>M & F	200 mg Cannabis sativa<br>5 min inhalation/day<br>GD 5.5–17.5	↓ BW	↑ Placental Weight<br>↓ Fetal:placental weight (M)	N/A	N/A	N/A
(Chang et al., 2017)	Mice<br>N/A	Δ9–THC<br>5 mg/kg/day (i.p.)<br>GD 5.5–18.5	↓ BW<br>↓ Litter size	↓ Trophoblast motility<br>↑ Diameters of trophoblastic septa<br>↓ Number of glycogen cells in junctional zone	N/A	N/A	N/A
(Natale et al., 2020)	Rats<br>M & F	Δ9–THC<br>3 mg/kg/day (i.p.)<br>GD 6.5–22	↓ BW<br>↓ Liver:BW<br>↓ Brain:BW	↑ Placental Weight<br>↓ Fetal:placental weight (M)<br>↑Placental labyrinth area<br>↓ *Epcam* expression<br>↓ Fetal blood space<br>↑ Pericytes recruitment<br>↓ Labyrinth trophoblast GLUT1 & GR	N/A	N/A	N/A

(Costa et al., 2015)	Cultured human cytotrophoblasts	∆9–THC<br>1-75 µM	N/A	↓ Trophoblast remodeling	N/A	N/A	N/A
(Gillies et al., 2020)	Rats<br>M & F	∆9–THC<br>3 mg/kg/day (i.p.)<br>GD 6–22	↓ BW		↓ Pancreas weight<br>↑ Glucose intolerance (at 5 mos; F)<br>↓ Pancreatic total and small islet density (at PND21 & 5 mos; F)<br>↓ β-cell mass (at 5 mos; F)	N/A	N/A
(Athanasiou et al., 2007)	Rat heart mitochondria	∆9–THC<br>0-200 µM	N/A	N/A	↓ Oxygen consumption & membrane potential of heart mitochondria	N/A	N/A
(Fišar et al., 2014)	Pig brain cortex mitochondria	∆9–THC<br>0-200 µM	N/A	N/A	↓ Mitochondria respiratory rate	N/A	N/A
(Jimenez-Blasco et al., 2020)	Mouse (CB_1 knockout)<br>M	∆9–THC<br>10 mg/kg challenge (i.p.)<br>24h prior to experiment	N/A	N/A	↓ Brain glucose to lactate conversion	No effect on prepulse inhibition, OFT, or EPM	N/A
(Lojpur et al., 2019)	Human BeWo trophoblasts	∆9–THC<br>3-30 µM	N/A	N/A	↓ Mitochondria respiration<br>↑ ER stress	N/A	N/A

(Breit et al., 2020)	Rats<br>M & F	∆9–THC<br>30 min Inhalation/day (100 mg/mL at 2 L/min airflow)<br>GD 5–20	No effect on BW	N/A	↓ Body weight at PND30	N/A	N/A
(Miranda et al., 2020)	Human induced pluripotent stem cells (hiPSC)	∆9–THC<br>10 µM<br>CBD<br>10 µM	N/A	N/A	N/A	∆9–THC: Precocious neuronal and glial differentiation<br>Abnormal functioning of voltage-gated calcium channels<br>CBD: neurotoxic effects	N/A
(Tortoriello et al., 2014)	Mice<br>M	∆9–THC<br>3 mg/kg/day (i.p.)<br>GD 5.5–17.5	N/A	N/A	N/A	↓ Fetal cortical and hippocampal connectivity	N/A
(Vargish et al., 2017)	Mice<br>M	∆9–THC<br>5 mg/kg/day (i.p.)<br>GD 10.5–18.5	N/A	N/A	N/A	↓ cholecystokinin interneurons in the hippocampus	N/A
(Mereu et al., 2003)	Rats<br>M	WIN (CB_1R agonist)<br>0.5 mg/kg/day (s.c.)<br>GD 5–20	N/A	N/A	N/A	↓ Long-term memory (passive avoidance task)<br>↓ LTP & Glutamate release in hippocampus	N/A
(Antonelli et al., 2004)	Rats<br>M	WIN (CB_1R agonist)	N/A	N/A	N/A	↓ Glutamate release in PFC	N/A

		0.5 mg/kg/day (s.c.)<br>GD 5–20					
(Antonelli et al., 2005)	Rats<br>M	WIN (CB_1R agonist)<br>0.5 mg/kg/day (s.c.)<br>GD 5–20	N/A	N/A	N/A	↓ Learning (homing behaviour & active avoidance task)<br>↓ Glutamate release in cortical cell cultures<br>↓ Cortical neuronal population	↓ Emotional Reactivity (separation-induced ultrasonic emission)
(Campolongo et al., 2007)	Rats<br>M	Δ9–THC<br>5 mg/kg/day (p.o.)<br>GD 15 – PND 9	No effect on BW	N/A	N/A	↓ Glutamate & norepinephrine in PFC<br>↓ Long-term memory (inhibitory avoidance task)<br>↓ Short-term olfactory memory (social discrimination task)	N/A
(Silva et al., 2012)	Rats<br>M & F	Δ9–THC<br>0.15 mg/kg/day (i.v.)<br>GD 1–21	No effect on BW	N/A	N/A	↓ Long-term memory (passive avoidance task)<br>↓ Spatial working memory (active place avoidance task) (M only)	↓ Locomotor response to amphetamine

						↓ Attention (attention task)	
(Beggiato et al., 2017)	Rats<br>M	∆9–THC<br>5 mg/kg/day (p.o.)<br>GD 15 – PND 9	No effect on BW	N/A	N/A	↓ GABA outflow and uptake in hippocampus slices	N/A
(Beggiato et al., 2020)	Rats<br>M	∆9–THC<br>5 mg/kg/day (p.o.)<br>GD 5–20	N/A	N/A	N/A	↓ Short-term memory (Y-maze)<br>↑ Kynurenine in PFC<br>↓ Glutamate in PFC	N/A
(Castaldo et al., 2007)	Rats<br>M	∆9–THC<br>5 mg/kg/day (i.p.)<br>WIN (CB_1R agonist)<br>0.5 mg/kg/day (p.o.)<br>GD 5–20	N/A	N/A	N/A	↓ Glutamate in PFC (∆9–THC & WIN)<br>↑ GLT1 & EAAC1 in PFC (WIN only)	N/A
(Castaldo et al., 2010)	Rats<br>M	∆9–THC<br>5 mg/kg/day (p.o.)<br>GD 15 – PND 9	N/A	N/A	N/A	↓ Glutamate outflow and uptake in hippocampus slices<br>↓ GLUT1 & GLAST in hippocampal slices	N/A
(Rubio et al., 1995)	Rats<br>M & F	∆9–THC<br>5 mg/kg/day (p.o.)	N/A	N/A	N/A	N/A	↑ Preference for morphine (place preference test), esp. in M

		GD 5 – PND 24					↑ Hypothalamic CRF & plasma CORT (F)<br>↓ Hypothalamic CRF & plasma CORT (M)
(Trezza et al., 2008)	Rats<br>M	∆9–THC<br>5 mg/kg/day (p.o.)<br>GD 15 – PND 9	No effect on BW	N/A	N/A	N/A	↑ Ultrasonic vocalizations<br>↑ Anxiety-like behaviour (EPM)<br>↓ Play behaviour and social interaction
(Newsom and Kelly, 2008)	Rats<br>M	∆9–THC<br>2 mg/kg/day (s.c.)<br>GD 1–22<br>PND 2–10	No effect on BW	N/A	N/A	N/A	↑ Anxiety-like behaviour (OFT)<br>↑ Social interaction
(Vela et al., 1998)	Rats<br>M & F	∆9–THC<br>5 mg/kg/day (p.o.)<br>GD 5 – PND 24	N/A	N/A	N/A	N/A	↑ Morphine self-administration (F)<br>↑ µ opioid receptor density in mesocorticolimbic brain regions (F)
(Moreno et al., 2003)	Rats<br>M & F	∆9–THC<br>0.1-2 mg/kg/day (p.o.)<br>GD 5 – PND 24	N/A	N/A	N/A	N/A	↑ Locomotor response to morphine, esp. in M

Summary of clinical and preclinical studies discussed in this review. Clinical studies discussed independently in the text that were also part of one or more of the meta-analyses or reviews are not independently highlighted in this table as they are already imbedded in the summary of the meta-analyse or review in which they are included. Only reviews that included a synthesis of studies (qualitative or quantitative) are included in this table. ∆9–THC: (-)-∆9– tetrahydrocannabinol; ASD: autism spectrum disorder; ASQ-SE: Ages and Stages: Social–Emotional Questionnaire;

BSID: Bayley Scales of Infant Development; BW: birth weight; CBCL: Child Behavior Checklist; CBD: cannabidiol; CORT: corticosterone; CRF: corticotropin releasing factor; D_2: dopamine receptor subtype D_2; EAAC1: excitatory amino acid carrier 1; EEG: electroencephalogram; EPM: elevated plus maze; ER: endoplasmic reticulum; F: female; fMRI: functional magnetic resonance imaging; GA: gestational age; GABA: γ-aminobutyric acid; GD: gestational day; GLAST: glutamate/aspartate transporter; GLUT1; glutamate transporter 1; GR: glucocorticoid receptor; i.p.: intraperitoneal; LTP: long-term potentiation; M: male; NICU: neonatal intensive care unit; OFT: open field test; PFC: prefrontal cortex; PLEs: psychosis-like events; PND: postnatal day; p.o.: per os (oral); PTD: preterm delivery; s.c.: subcutaneous; SDSC: Sleep Disturbance Scale for Children; SGA: small for gestational age. Result summaries for OPPS, MHPCD, and GenR cohorts adapted from McLemore & Richardson, 2016.

Table References

Antonelli, T., Tanganelli, S., Tomasini, M.C., Finetti, S., Trabace, L., Steardo, L., Sabino, V., Carratu, M.R., Cuomo, V., and Ferraro, L. (2004). Long-term effects on cortical glutamate release induced by prenatal exposure to the cannabinoid receptor agonist (R)-(+)-[2,3-dihydro-5-methyl-3-(4-morpholinyl-methyl)pyrrolo[1,2,3-de]-1,4-benzo xazin-6-yl]-1-naphthalenylmethanone: an in vivo microdialysis study in the awake rat. *Neuroscience* 124**,** 367-375.

Antonelli, T., Tomasini, M.C., Tattoli, M., Cassano, T., Tanganelli, S., Finetti, S., Mazzoni, E., Trabace, L., Steardo, L., Cuomo, V., and Ferraro, L. (2005). Prenatal exposure to the CB1 receptor agonist WIN 55,212-2 causes learning disruption associated with impaired cortical NMDA receptor function and emotional reactivity changes in rat offspring. *Cereb Cortex* 15**,** 2013-2020.

Athanasiou, A., Clarke, A.B., Turner, A.E., Kumaran, N.M., Vakilpour, S., Smith, P.A., Bagiokou, D., Bradshaw, T.D., Westwell, A.D., Fang, L., Lobo, D.N., Constantinescu, C.S., Calabrese, V., Loesch, A., Alexander, S.P., Clothier, R.H., Kendall, D.A., and Bates, T.E. (2007). Cannabinoid receptor agonists are mitochondrial inhibitors: a unified hypothesis of how cannabinoids modulate mitochondrial function and induce cell death. *Biochem Biophys Res Commun* 364**,** 131-137.

Bailey, B.A., Wood, D.L., and Shah, D. (2020). Impact of pregnancy marijuana use on birth outcomes: results from two matched population-based cohorts. *J Perinatol*.

Beggiato, S., Borelli, A.C., Tomasini, M.C., Morgano, L., Antonelli, T., Tanganelli, S., Cuomo, V., and Ferraro, L. (2017). Long-lasting alterations of hippocampal GABAergic neurotransmission in adult rats following perinatal Delta(9)-THC exposure. *Neurobiol Learn Mem* 139**,** 135-143.

Beggiato, S., Ieraci, A., Tomasini, M.C., Schwarcz, R., and Ferraro, L. (2020). Prenatal THC exposure raises kynurenic acid levels in the prefrontal cortex of adult rats. *Prog Neuropsychopharmacol Biol Psychiatry* 100**,** 109883.

Benevenuto, S.G., Domenico, M.D., Martins, M.A., Costa, N.S., De Souza, A.R., Costa, J.L., Tavares, M.F., Dolhnikoff, M., and Veras, M.M. (2017). Recreational use of marijuana during pregnancy and negative gestational and fetal outcomes: An experimental study in mice. *Toxicology* 376**,** 94-101.

Bolhuis, K., Kushner, S.A., Yalniz, S., Hillegers, M.H.J., Jaddoe, V.W.V., Tiemeier, H., and El Marroun, H. (2018). Maternal and paternal cannabis use during pregnancy and the risk of psychotic-like experiences in the offspring. *Schizophr Res* 202**,** 322-327.

Breit, K.R., Rodriguez, C., Lei, A., and Thomas, J.D. (2020). Combined vapor exposure to THC and alcohol in pregnant rats: Maternal outcomes and pharmacokinetic effects. *Neurotoxicol Teratol***,** 106930.

Campolongo, P., Trezza, V., Cassano, T., Gaetani, S., Morgese, M.G., Ubaldi, M., Soverchia, L., Antonelli, T., Ferraro, L., Massi, M., Ciccocioppo, R., and Cuomo, V. (2007). Perinatal exposure to delta-9-tetrahydrocannabinol causes enduring cognitive deficits associated with alteration of cortical gene expression and neurotransmission in rats. *Addict Biol* 12**,** 485-495.

Carter, R.C., Wainwright, H., Molteno, C.D., Georgieff, M.K., Dodge, N.C., Warton, F., Meintjes, E.M., Jacobson, J.L., and Jacobson, S.W. (2016). Alcohol, Methamphetamine, and Marijuana Exposure Have Distinct Effects on the Human Placenta. *Alcohol Clin Exp Res* 40**,** 753-764.

Castaldo, P., Magi, S., Cataldi, M., Arcangeli, S., Lariccia, V., Nasti, A.A., Ferraro, L., Tomasini, M.C., Antonelli, T., Cassano, T., Cuomo, V., and Amoroso, S. (2010). Altered regulation of glutamate release and decreased functional activity and expression of GLT1 and GLAST glutamate transporters in the hippocampus of adolescent rats perinatally exposed to Delta(9)-THC. *Pharmacol Res* 61**,** 334-341.

Castaldo, P., Magi, S., Gaetani, S., Cassano, T., Ferraro, L., Antonelli, T., Amoroso, S., and Cuomo, V. (2007). Prenatal exposure to the cannabinoid receptor agonist WIN 55,212-2 increases glutamate uptake through overexpression of GLT1 and EAAC1 glutamate transporter subtypes in rat frontal cerebral cortex. *Neuropharmacology* 53**,** 369-378.

Chang, X., Bian, Y., He, Q., Yao, J., Zhu, J., Wu, J., Wang, K., and Duan, T. (2017). Suppression of STAT3 Signaling by Delta9-Tetrahydrocannabinol (THC) Induces Trophoblast Dysfunction. *Cell Physiol Biochem* 42**,** 537-550.

Conner, S.N., Bedell, V., Lipsey, K., Macones, G.A., Cahill, A.G., and Tuuli, M.G. (2016). Maternal Marijuana Use and Adverse Neonatal Outcomes: A Systematic Review and Meta-analysis. *Obstet Gynecol* 128**,** 713-723.

Corsi, D.J., Donelle, J., Sucha, E., Hawken, S., Hsu, H., El-Chaar, D., Bisnaire, L., Fell, D., Wen, S.W., and Walker, M. (2020). Maternal cannabis use in pregnancy and child neurodevelopmental outcomes. *Nat Med* 26**,** 1536-1540.

Costa, M.A., Fonseca, B.M., Marques, F., Teixeira, N.A., and Correia-Da-Silva, G. (2015). The psychoactive compound of Cannabis sativa, Delta(9)-tetrahydrocannabinol (THC) inhibits the human trophoblast cell turnover. *Toxicology* 334**,** 94-103.

Dahl, R.E., Scher, M.S., Williamson, D.E., Robles, N., and Day, N. (1995). A longitudinal study of prenatal marijuana use. Effects on sleep and arousal at age 3 years. *Arch Pediatr Adolesc Med* 149**,** 145-150.

Day, N., Sambamoorthi, U., Taylor, P., Richardson, G., Robles, N., Jhon, Y., Scher, M., Stoffer, D., Cornelius, M., and Jasperse, D. (1991). Prenatal marijuana use and neonatal outcome. *Neurotoxicology and Teratology* 13**,** 329-334.

Eiden, R.D., Shisler, S., Granger, D.A., Schuetze, P., Colangelo, J., and Huestis, M.A. (2020). Prenatal Tobacco and Cannabis Exposure: Associations with Cortisol Reactivity in Early School Age Children. *Int J Behav Med* 27**,** 343-356.

El Marroun, H., Bolhuis, K., Franken, I.H.A., Jaddoe, V.W.V., Hillegers, M.H., Lahey, B.B., and Tiemeier, H. (2019). Preconception and prenatal cannabis use and the risk of behavioural and emotional problems in the offspring; a multi-informant prospective longitudinal study. *Int J Epidemiol* 48**,** 287-296.

El Marroun, H., Tiemeier, H., Steegers, E.A., Jaddoe, V.W., Hofman, A., Verhulst, F.C., Van Den Brink, W., and Huizink, A.C. (2009). Intrauterine cannabis exposure affects fetal growth trajectories: the Generation R Study. *J Am Acad Child Adolesc Psychiatry* 48**,** 1173-1181.

English, D.R., Hulse Gk Fau - Milne, E., Milne E Fau - Holman, C.D., Holman Cd Fau - Bower, C.I., and Bower, C.I. (1997). Maternal cannabis use and birth weight: a meta-analysis. *Addiction* 92**,** 1553-1560.

Fine, J.D., Moreau, A.L., Karcher, N.R., Agrawal, A., Rogers, C.E., Barch, D.M., and Bogdan, R. (2019). Association of Prenatal Cannabis Exposure With Psychosis Proneness Among Children in the Adolescent Brain Cognitive Development (ABCD) Study. *JAMA Psychiatry* 76**,** 762-764.

Fišar, Z., Singh, N., and Hroudová, J. (2014). Cannabinoid-induced changes in respiration of brain mitochondria. *Toxicol Lett* 231**,** 62-71.

Fried, P.A. (1980). Marihuana use by pregnant women: Neurobehavioral effects in neonates. *Drug and Alcohol Dependence* 6**,** 415-424.

Fried, P.A., and Watkinson, B. (1988). 12- and 24-month neurobehavioural follow-up of children prenatally exposed to marihuana, cigarettes and alcohol. *Neurotoxicol Teratol* 10**,** 305-313.

Fried, P.A., Watkinson, B., and Gray, R. (1998). Differential effects on cognitive functioning in 9- to 12-year olds prenatally exposed to cigarettes and marihuana. *Neurotoxicol Teratol* 20**,** 293-306.

Gillies, R., Lee, K., Vanin, S., Laviolette, S.R., Holloway, A.C., Arany, E., and Hardy, D.B. (2020). Maternal exposure to Delta9-tetrahydrocannabinol impairs female offspring glucose homeostasis and endocrine pancreatic development in the rat. *Reprod Toxicol* 94**,** 84-91.

Goldschmidt, L., Richardson, G.A., Cornelius, M.D., and Day, N.L. (2004). Prenatal marijuana and alcohol exposure and academic achievement at age 10. *Neurotoxicol Teratol* 26**,** 521-532.

Gunn, J.K., Rosales, C.B., Center, K.E., Nunez, A., Gibson, S.J., Christ, C., and Ehiri, J.E. (2016). Prenatal exposure to cannabis and maternal and child health outcomes: a systematic review and meta-analysis. *BMJ Open* 6**,** e009986.

Jaddoe, V.W., Van Duijn, C.M., Franco, O.H., Van Der Heijden, A.J., Van Iizendoorn, M.H., De Jongste, J.C., Van Der Lugt, A., Mackenbach, J.P., Moll, H.A., Raat, H., Rivadeneira, F., Steegers, E.A., Tiemeier, H., Uitterlinden, A.G., Verhulst, F.C., and Hofman, A. (2012). The Generation R Study: design and cohort update 2012. *Eur J Epidemiol* 27**,** 739-756.

Jimenez-Blasco, D., Busquets-Garcia, A., Hebert-Chatelain, E., Serrat, R., Vicente-Gutierrez, C., Ioannidou, C., Gomez-Sotres, P., Lopez-Fabuel, I., Resch-Beusher, M., Resel, E., Arnouil, D., Saraswat, D., Varilh, M., Cannich, A., Julio-Kalajzic, F., Bonilla-Del Rio, I., Almeida, A., Puente, N., Achicallende, S., Lopez-Rodriguez, M.L., Jolle, C., Deglon, N., Pellerin, L., Josephine, C., Bonvento, G., Panatier, A., Lutz, B., Piazza, P.V., Guzman, M., Bellocchio, L., Bouzier-Sore, A.K., Grandes, P., Bolanos, J.P., and Marsicano, G. (2020). Glucose metabolism links astroglial mitochondria to cannabinoid effects. *Nature* 583**,** 603-608.

Kharbanda, E.O., Vazquez-Benitez, G., Kunin-Batson, A., Nordin, J.D., Olsen, A., and Romitti, P.A. (2020). Birth and early developmental screening outcomes associated with cannabis exposure during pregnancy. *J Perinatol*.

Lojpur, T., Easton, Z., Raez-Villanueva, S., Laviolette, S., Holloway, A.C., and Hardy, D.B. (2019). Δ9-Tetrahydrocannabinol leads to endoplasmic reticulum stress and mitochondrial dysfunction in human BeWo trophoblasts. *Reprod Toxicol* 87**,** 21-31.

Mereu, G., Fa, M., Ferraro, L., Cagiano, R., Antonelli, T., Tattoli, M., Ghiglieri, V., Tanganelli, S., Gessa, G.L., and Cuomo, V. (2003). Prenatal exposure to a cannabinoid agonist produces memory deficits linked to dysfunction in hippocampal long-term potentiation and glutamate release. *Proc Natl Acad Sci U S A* 100**,** 4915-4920.

Miranda, C.C., Barata, T., Vaz, S.H., Ferreira, C., Quintas, A., and Bekman, E.P. (2020). hiPSC-Based Model of Prenatal Exposure to Cannabinoids: Effect on Neuronal Differentiation. *Front Mol Neurosci* 13**,** 119.

Moreno, M., Trigo, J.M., Escuredo, L., RodríGuez De Fonseca, F., and Navarro, M. (2003). Perinatal exposure to Δ9-tetrahydrocannabinol increases presynaptic dopamine D2 receptor sensitivity: a behavioral study in rats. *Pharmacology Biochemistry and Behavior* 75**,** 565-575.

Natale, B.V., Gustin, K.N., Lee, K., Holloway, A.C., Laviolette, S.R., Natale, D.R.C., and Hardy, D.B. (2020). Δ9-tetrahydrocannabinol exposure during rat pregnancy leads to symmetrical fetal growth restriction and labyrinth-specific vascular defects in the placenta. *Scientific Reports* 10.

Nawa, N., Garrison-Desany, H.M., Kim, Y., Ji, Y., Hong, X., Wang, G., Pearson, C., Zuckerman, B.S., Wang, X., and Surkan, P.J. (2020). Maternal persistent marijuana use and cigarette smoking are independently associated with shorter gestational age. *Paediatr Perinat Epidemiol*.

Newsom, R.J., and Kelly, S.J. (2008). Perinatal delta-9-tetrahydrocannabinol exposure disrupts social and open field behavior in adult male rats. *Neurotoxicol Teratol* 30**,** 213-219.

Ortigosa, S., Friguls, B., Joya, X., Martinez, S., Marinoso, M.L., Alameda, F., Vall, O., and Garcia-Algar, O. (2012). Feto-placental morphological effects of prenatal exposure to drugs of abuse. *Reprod Toxicol* 34**,** 73-79.

Paul, S.E., Hatoum, A.S., Fine, J.D., Johnson, E.C., Hansen, I., Karcher, N.R., Moreau, A.L., Bondy, E., Qu, Y., Carter, E.B., Rogers, C.E., Agrawal, A., Barch, D.M., and Bogdan, R. (2020). Associations Between Prenatal Cannabis Exposure and Childhood Outcomes: Results From the ABCD Study. *JAMA Psychiatry*.

Rubio, P., Rodriguez De Fonseca, F., Munoz, R.M., Ariznavarreta, C., Martin-Calderon, J.L., and Navarro, M. (1995). Long-term behavioral effects of perinatal exposure to delta 9-tetrahydrocannabinol in rats: possible role of pituitary-adrenal axis. *Life Sci* 56**,** 2169-2176.

Scher, M.S., Richardson, G.A., Coble, P.A., Day, N.L., and Stoffer, D.S. (1988). The effects of prenatal alcohol and marijuana exposure: disturbances in neonatal sleep cycling and arousal. *Pediatr Res* 24**,** 101-105.

Silva, L., Zhao, N., Popp, S., and Dow-Edwards, D. (2012). Prenatal tetrahydrocannabinol (THC) alters cognitive function and amphetamine response from weaning to adulthood in the rat. *Neurotoxicol Teratol* 34**,** 63-71.

Singh, S., Filion, K.B., Abenhaim, H.A., and Eisenberg, M.J. (2020). Prevalence and outcomes of prenatal recreational cannabis use in high-income countries: a scoping review. *BJOG* 127**,** 8-16.

Smith, A.M., Fried, P.A., Hogan, M.J., and Cameron, I. (2006). Effects of prenatal marijuana on visuospatial working memory: an fMRI study in young adults. *Neurotoxicol Teratol* 28**,** 286-295.

Smith, A.M., Mioduszewski, O., Hatchard, T., Byron-Alhassan, A., Fall, C., and Fried, P.A. (2016). Prenatal marijuana exposure impacts executive functioning into young adulthood: An fMRI study. *Neurotoxicol Teratol* 58**,** 53-59.

Stroud, L.R., Papandonatos, G.D., Jao, N.C., Vergara-Lopez, C., Huestis, M.A., and Salisbury, A.L. (2020). Prenatal tobacco and marijuana co-use: Sex-specific influences on infant cortisol stress response. *Neurotoxicol Teratol* 79**,** 106882.

Tortoriello, G., Morris, C.V., Alpar, A., Fuzik, J., Shirran, S.L., Calvigioni, D., Keimpema, E., Botting, C.H., Reinecke, K., Herdegen, T., Courtney, M., Hurd, Y.L., and Harkany, T. (2014). Miswiring the brain: Delta9-tetrahydrocannabinol disrupts cortical development by inducing an SCG10/stathmin-2 degradation pathway. *EMBO J* 33**,** 668-685.

Trezza, V., Campolongo, P., Cassano, T., Macheda, T., Dipasquale, P., Carratu, M.R., Gaetani, S., and Cuomo, V. (2008). Effects of perinatal exposure to delta-9-tetrahydrocannabinol on the emotional reactivity of the offspring: a longitudinal behavioral study in Wistar rats. *Psychopharmacology (Berl)* 198**,** 529-537.

Vargish, G.A., Pelkey, K.A., Yuan, X., Chittajallu, R., Collins, D., Fang, C., and Mcbain, C.J. (2017). Persistent inhibitory circuit defects and disrupted social behaviour following in utero exogenous cannabinoid exposure. *Mol Psychiatry* 22**,** 56-67.

Vela, G., Martin, S., Garcia-Gil, L., Crespo, J.A., Ruiz-Gayo, M., Fernandez-Ruiz, J.J., Garcia-Lecumberri, C., Pelaprat, D., Fuentes, J.A., Ramos, J.A., and Ambrosio, E. (1998). Maternal exposure to delta9-tetrahydrocannabinol facilitates morphine self-administration behavior and changes regional binding to central mu opioid receptors in adult offspring female rats. *Brain Res* 807**,** 101-109.

Wang, X., Dow-Edwards, D., Anderson, V., Minkoff, H., and Hurd, Y.L. (2004). In utero marijuana exposure associated with abnormal amygdala dopamine D2 gene expression in the human fetus. *Biol Psychiatry* 56**,** 909-915.

Winiger, E.A., and Hewitt, J.K. (2020). Prenatal cannabis exposure and sleep outcomes in children 9-10 years of age in the adolescent brain cognitive development (SM) study. *Sleep Health*.
